# Detection of Legionella Pneumophila in Urine and Serum Specimens of Neutropenic Febrile Patients with Haematological Malignancies

**Published:** 2017-01-01

**Authors:** Nastaran Farzi, Zahra Abrehdari-Tafreshi, Omid Zarei, Leili Chamani-Tabriz

**Affiliations:** 1 Department of Microbiology, Islamic Azad University, North Tehran Branch, Tehran, Iran; 2 Department of Biology, Islamic Azad University, Science and Research Branch, Tehran, Iran; 3 Biotechnology Research Center, Tabriz University of Medical Sciences, Tabriz, Iran; 4 Reproductive Biotechnology Research Center, Avicenna Research Institute (ACECR), Tehran, Iran

**Keywords:** Legionella pneumophila, Malignancy, Mip, Neutropenia

## Abstract

**Background: **Legionella pneumophila (L. pneumophila) is a gram-negative bacterium which causes ‎Legionnaires’ disease as well as Pontiac fever. The Legionella infections in patients suffering from ‎neutropenia- as a common complication of cancer chemotherapy- can distribute rapidly. We ‎aimed to detect of L. pneumophila in haematological malignancy suffering patients with ‎neutropenic fever by targeting the (macrophage infectivity potentiator) mip gene.

**Subjects and** **Methods:** Serum and ‎urine specimens were obtained from 80 patients and presence of mip gene of L. pneumophila in ‎specimens was investigated by PCR.

**Results:** The L. pneumophila infection was detected in 21 (26.2%) and 38 ‎‎(47.5%) of urine and serum specimens, respectively.

**Conclusion:** Our findings indicated that the relative high ‎prevalence of L. pneumophila in the studied patients group which show the necessity of ‎considering this microorganism in future studies from detection and treatment point of view in ‎cancer patients.

## Introduction

 Legionella pneumophila (L. pneumophila) is a fastidious, gram-negative and facultative intracellular bacterium. It is the causative agent of Legionnaires’ disease- a severe form of pneumonia- also causes the Pontiac fever which includes a flu-like, self-limiting illness. This micro organism mostly exists in freshwater environments but can also inhabit in man-made water distribution systems, which are the main sources of infection.^1^ Since the first description of Legionnaires’ disease in 1976, L. pneumophila infections have become a significant cause of hospital-acquired morbidity and mortality.^2^ The most important ways of transmission of L. pneumophila infections are through inhalation of the contaminated aerosols which can follow by thereplication of bacteria within human alveolar macrophages.^3^ The infection in patients suffering from severe neutropenia can distribute rapidly.^4^ Beside the numerous reports regarding to introduce the risk factors to develop of L. pneumophila infections, the severe neutropenia which is defined as absolute neutrophil count ≤500/mm^3^,^5^^,^^6^ and also is an important complication in cancer patients, remains as most common predisposing factor for L. pneumophila infections in such patients.^7^ The early detection of this infection in cancer patients is critical and delaying in appropriate therapy increases the mortality rate.^8^^,^^9^ Among the several introduced methods for L. pneumophila detection, the polymerase chain reaction (PCR) have been utilized extensively for L. pneumophila detection in respiratory secretions,^10^ urine^11^ and serum^12^ as well environmental samples,^13^ also the (macrophage infectivity potentiator) mip gene has been targeted in nucleic acid amplification-based methods for identification of this microorganism.^14^^,^^15^ Despite the existence plenty of reports related to L. pneumophila detection in different groups of patients,^15^^-^^17^ the number of studies regarding to prevalence the this microorganism in neutropenic fever patients with haematological malignancy is not more. Therefore, we aimed to find out the presence of L. pneumophila in urine and serum specimens of this patients group using PCR by targeting mip gene in order to show the importance of this microorganism in such patients and emphasis to design more strategies in hospital environments to prevent of L. pneumophila infections.

## SUBJECTS AND METHODS

**Samples collection and DNA extraction**

Eighty patients with hospitalized haematological malignancy and confirmed neutropenic fever (granulocyte count of <1,000/mm^3^) in Imam Khomeini and Taleghani hospitals, Tehran, Iran (with age range: 14-80 years old) presented between June 2013 to July 2014, were included in this study. Informed consent and besides Information were obtained from all participants. (Ethical code: 89/1412 from Avicenna Research Institute)

Five mL peripheral blood and 15 mL urine specimens were obtained from each patient. The samples were stored at -70**°**C until analysis**. **The bacterial DNA extracted from serum and urine samples by Genomic DNA Extraction Kit (Bioneer, Seoul, South Korea) according to manufacture instruction.

**PCR assay**

The extracted DNA from serum and urine specimens were subjected to PCR of mip gene in the presence of standard PCR mixture containing 20 mM Tris/HCl, pH 8.3, 100 mM KCl, 1.5 mM MgCl_2, _0.25 mM dNTP and 10 pM of each primer and 1U Taq DNA polymerase. The used primers in this study

were as follows: Forward primer sequence: 5´-GCT TTA ACC GAA CAG CAA ATG-3´ and Reverse primer sequence: 5´-AAC GGT ACC ATC AAT CAG ACG-3´which made a PCR product of 267 bp.^18^ The cycling conditions of amplification were as follows: initial denaturation at 95^°^C for 5 min followed by 37 cycles of denaturation at 95°C for 1 min, annealing at 58^°^C for 30 sec, extension at 72^°^C for 30 sec and final extension at 72°C for 5 min. The products of PCR were electrophoresed through 1.5% agarose (Promega Co., USA) gel at 80 V for 1 to 1:30 h in TAE buffer. Ethidium bromide 0.4 mg/ml (Sigma Chemicals Co, USA) was added to the gel to visualize DNA on UV transilluminator.

## Results

 Urine and serum specimens from onco-haematological patients including acute myeloid leukemia (AML), acute lymphoid leukemia (ALL), multiple myeloma (MM), Hodgkin lymphoma (HL), non-Hodgkin lymphoma (NHL) and hairy cell leukemia (HCL) with confirmed febrile neutropenia were examined for presence of mip gene of L. pneumophila by PCR. The data showed the presence of L. pneumophila in 21 (26.2%) and 38 (47.5%) of urine and serum specimens, respectively from 80 patients. The numbers of positive cases in serum samples were higher than in urine (except for MM patients). All positive urine cases were positive in serum analysis. The characteristics of L. pneumophila-infected patients have been summarized in Table 1.

**Table 1:** Characteristics of Legionella pneumophila-infected patients with haematological malignancies and neutropenic fever

**Type**	**Patients**	**Serum ** **Positive**	**Urine ** **Positive**	**Both ** **specimens ** **Positive**
**ALL**	16	10	8	8
**AML**	33	13	8	8
**HL**	14	9	1	1
**NHL**	7	2	0	0
**MM**	7	4	4	4
**HCL**	3	0	0	0
**Total**	80 (100%)	38 (47.5%)	21 (26.2%)	21 (26.2%)

## Discussion

 Infection remains a major problem in cancer patients and neutropenic fever as a common complication of anti neoplastic chemotherapy make these patients more susceptible for infections.^20^^,^^21^ Among several bacterial agents which have been detected in cancer patients,^22^ L. pneumophila ‎is considered as an important cause of infections in such patients.^7^ Here, we designed our study to detect of the mip gene of L. pneumophila in urine and serum samples of neutropenic febrile patients with haematological malignancies by PCR. We detected the mip gene of L. pneumophila in 21 (26.2%) and 38 (47.5%) in urine and serum of 80 patients, respectively. This findings are demonstrative the importance of L. pneumophila in this group of patient which support the previous studies regarding to detection of this microorganism in cancer patients.^7^^,^^19^^,^^23^

Different diagnostic methods include serological testing, direct fluorescent antibody staining (DFA), the urinary antigen detection and PCR-based techniques have been developed for detection of L. pneumophila infections.^24^ Detection of antibody levels against L. pneumophila in serum is not suitable diagnosis method during the acute phase of disease. DFA on respiratory specimens is a rapid method but it has low sensitivity.^25^ Commercially urinary antigen assays also are available and have been shown that can be useful for L. pneumophila decetion.^26^^,^^27^ Although the bacterial culture remains as “gold standard” method for this purpose, but due to the relatively slow growing (3-10 days) and fastidious nature of this microorganism,^24^^,^^28^ also decrease sensitivity to culture in specimens derived from treated patients with a broad-spectrum antibiotic,^29^ the other strategies to ensure a rapid diagnosis of legionellosis have become imperative. The PCR-based techniques have been shown that can be promising method for the rapid diagnosis of L. pneumophila particularly in culture and serum antibody negative individuals.^30^ These techniques have provided acceptable sensitivity,^24^^,^^31^^,^^32^ therefore, we used PCR in our study for detection of this microorganism using urine and serum specimens. These specimens have been utilized extensively for L. pneumophila detection in infected patients.^5^^,^^33^ These non-respiratory specimens seem more suitable than sputum for identification of L. pneumophila because it has been shown that fewer than half of the Legionnaires’ disease patients produce sputum.

In our study, the numbers of positive cases in urine were lower than the serum samples (21 cases for urine vs. 38 cases for serum from 80 patients). Although in ALL patients, number of positive cases for L. pneumophila by examination of urine and serum has no very difference (10 and 8 cases for serum and urine, respectively) also in MM suffering individuals the positive cases in both urine and serum specimens are equal but overall our findings suggest that urine sample cannot be suitable for detection of L. pneumophila by PCR. It should be note that this findings must be interpret by caution because it is in contrast with some investigations which have been reported a high detection rate of Legionella DNA in urine,^5^^,^^34^^,^^35^ In our work we did not have bacterial culture equipment to evaluate this differences between urine and serum specimens but at least there is one study that confirms our data about unsuitableness of urine samples for identification of L. pneumophila by PCR.^36^

Although number of patients was not large and we did not perform the bacterial culture, similar to other reports^37^^,^^38^ but the present data showed relative high prevalence of L. pneumophila in haematological malignancy patients with confirmed neutropenic fever which highlights the importance of re-planning to prevent this kind of infections in cancer care unit environment. Furthermore, acquired data are suggestive the PCR as suitable and rapid method for detection of L. pneumophila, however more studies should be sought to judgment between suitableness of urine and serum specimens. Also, the association between underlying disorders such as chronic renal failure, hepatic cirrhosis, diabetes, heart disease, alcoholism, smoking and addiction with susceptibility to L. pneumophila infection in these patients should be evaluated in future studies.

## CONCLUSION

 Our findings indicated the relative high prevalence of L. pneumophila in neutropenic febrile patients with haematological malignancies which shows the necessity of considering this microorganism in future studies from detection and treatment point of view in cancer patients.
